# Detection and identification of factors in the atrium responsible for blood pressure regulation in patients with hypertension

**DOI:** 10.1007/s00380-024-02362-0

**Published:** 2024-03-07

**Authors:** Kenshi Yoshimura, Wei Mengyan, Shinichiro Kume, Tatsuki Kurokawa, Shinji Miyamoto, Yoichi Mizukami, Katsushige Ono

**Affiliations:** 1 Department of Pathophysiology, Oita University School of Medicine, 1-1 Idaigaoka, Hasama, Yufu, Oita 879-5593 Japan; 2 Department of Cardiovascular Surgery, Oita University School of Medicine, Oita University, Yufu, Oita Japan; 3 Institute of Gene Research, Yamaguchi University Science Research Center, Ube, Yamaguchi Japan

**Keywords:** Atrial appendectomy, Gene expression profile, Principal component analysis, Blood pressure

## Abstract

Resection of the left atrial appendage reportedly improves blood pressure in patients with hypertension. This study aimed to validate the transcriptional profiles of atrial genes responsible for blood pressure regulation in patients with hypertension as well as to identify the molecular mechanisms in rat biological systems. RNA sequencing data of left atrial appendages from patients with (*n* = 6) and without (*n* = 6) hypertension were subjected to unsupervised principal component analysis (PCA). Reduction of blood pressure was reflected by third and ninth principal components PC3 and PC9, and that eighteen transcripts, including endothelin-1, were revealed by PCA-based pathway analysis. Resection of the left atrial appendage in hypertensive rats improved their blood pressure accompanied by a decrease in serum endothelin-1 concentration. Expression of the endothelin-1 gene in the atrium and atrial appendectomy could play roles in blood pressure regulation in humans and rats.

## Introduction

Atrial fibrillation (AF) is one of the most common arrythmias [1, 2]. AF can cause critical or even fatal thrombosis, which may lead to cerebral infarction, visceral infarction, and/or acute limb ischemia. To decrease the risk of ischemic complications, patients with AF should receive long-term oral anticoagulation [1, 2]. Surgical resection or occlusion of the left atrial appendage (LAA) has been hypothesized to prevent ischemic stroke in patients with AF because LAA thrombi are believed to be the main source of cardiac thrombi. Although minimally invasive thoracoscopic atrial appendectomy is curative and may be safely performed even in patients with poor left ventricular function, there are several methods for percutaneous LAA closure, including the Watchman system and the Lariat device [3, 4]. Hypertension is a significant risk factor for AF, with both pathologies often coexisting. Thus, patients with AF who undergo LAA resection or exclusion also often suffer from hypertension. Interestingly, LAA exclusion (LAAE) using the Lariat device improves blood pressure in patients with AF and hypertension, whereas that using the Watchman device does not [5]. The suppression of the renin–angiotensin–aldosterone system and the reduction of catecholamine levels, such as noradrenaline and adrenaline, are involved in this phenomenon [5, 6]. However, the underlying mechanism for the changes in blood pressure after LAAE using the Lariat device remains unclear. Hence, in this study, we investigated the gene expression profiles in surgically resected LAA tissues of patients with AF who underwent open heart surgery. Additionally, using an animal model, we evaluated the role of endothelin-1 (ET-1) coded by *EDN1* as a potential factor responsible for blood pressure regulation.

## Materials and methods

### Sample collection

Patients who underwent open heart surgery and concomitant LAA resection from 2014 to 2021 at Oita University Hospital were enrolled in this study. The LAA of patients with AF was resected, cut into small pieces, and immersed into an RNAlater™ solution (QIAGEN N.V., Venlo, Limburg, Netherlands) within 5 min from resection to prevent RNA degeneration. Tissues were preserved at 4 °C for one night and thereafter preserved at − 80 °C until further analysis. Written informed consent was obtained from all participants. This study was approved by the institutional review board of Oita University Hospital (approval number: P-14-03).

### RNA sequencing (RNA-Seq) of the LAA

We selected 12 participants according to the following criteria: male, aged 50–80 years, with chronic AF, and never diagnosed with thyroid disease, diabetes mellitus, chronic kidney disease, or diseases that require steroid administration. The selected participants were divided into the hypertension (*n* = 6) and normotension (*n* = 6) groups. LAA tissues of the participants were thawed for total RNA extraction using the Maxwell RSC simplyRNA Tissue Kit (Promega, Madison, WI, USA). To evaluate the quality of the extracted RNA, the RNA Integrity Number (RIN) was checked using Bioanalyzer (Agilent Technologies, Inc. Santa Clara, CA, USA), with an RIN of > 7 considered as good quality and suitable for RNA sequencing. To create the RNA-Seq library, poly (A) RNA extraction and fragmentation were performed with 10 ng of total RNA using NEBNext Poly(A) mRNA Magnetic Isolation Module (New England Biolabs, Ipswich, MA, USA) and NEBNext Ultra II RNA Library Prep Kit for Illumina (New England Biolabs, Ipswich, MA, USA) following the instructions of the NEBNext Ultra II RNA Library Prep Kit. From this fragmented poly (A) RNA, complementary DNA (cDNA) was generated through reverse transcription, and an adaptor sequence was attached to the cDNA using NEBNext Adaptor (New England Biolabs, Ipswich, MA, USA). The RNA-Seq library was created by multiplying cDNA using polymerase chain reaction. The concentration and length of cDNA in the library of each sample were checked using Qubit (Thermo Fisher Scientific, Waltham, MA, USA) and Bioanalyzer, respectively. RNA-Seq analysis of each sample was performed using Illumina NextSeq 500 (Illumina, Inc., San Diego, CA, USA). CLC Genomics Workbench 20.0.4 (QIAGEN N. V., Venlo, Limburg, Netherlands) was used to manage the RNA-Seq data obtained from Illumina NextSeq 500, to trim reads in all samples to 75 bp, and to count reads mapped to a reference sequence. The reference sequence and genome annotation were downloaded from Homo sapience GRCh37 of Ensembl database (URL: https://asia.ensembl.org/index.html).

### Comparison between the two groups

The reads per kilobase of exon per million mapped reads (RPKM) and transcripts per million (TPM), which were values normalized by the length of each gene and by the total read amounts of each sample, were calculated using the following formula:

$$\mathrm{RPKM }= \frac{\mathrm{Total exon reads}}{\mathrm{mapped reads}\left({10}^{6}\right) \times \mathrm{ exon length}\left({10}^{3}\right)},$$

$$\mathrm{TPM }= \frac{\mathrm{RPKM }\times {10}^{6}}{\mathrm{\Sigma RPKM}}.$$

To compare the genes expression levels between the hypertension and normotension groups, we used log_2_ (TPM + 1) of each sample. Subtracting the average of log_2_ (TPM + 1) of the normotension group from that of the hypertension group obtained the log_2_ fold change. The differences in gene expression between the two groups were tested using Welch’s *t* test. A *p*-value of < 0.05 was considered statistically significant.

### Multivariate analysis

To clarify the gene expression tendency in each group, we performed multivariate analyses, including hierarchical clustering analysis and principal component analysis (PCA). In the multivariate analyses, we speculated the hypertension-specific genes expressed in the LAA. To identify important genes in the pathogenesis of hypertension, we selected specific genes from the results of the PCA that had principal component scores that were within the range of the same direction in which principal component vectors of hypertension group gathered. These genes were then included in the pathway analysis.

### Pathway analysis

From among the selected genes, those that met the threshold of log_2_ fold change > 0.5 were included in the pathway analysis using Ingenuity Pathway Analysis (IPA, QIAGEN N.V., Venlo, Limburg, Netherlands). Among the IPA results, we focused on results of diseases and functions, which was the downstream pathway analysis. We found that the hypertension ranked in the top 5 of all results of diseases and functions analyses. Eighteen genes were identified in this analysis, and we then performed STRING analysis (https://string-db.org/) to clarify the association among these genes.

### Experiment using rat model of LAAE

All animal experimental protocols were approved in advance by the Ethics Review Committee for Animal Experimentation of Oita University School of Medicine (No. 210401), and were carried out according to the guidelines for animal research of the Physiological Society of Japan to minimize the number of animals. Eight-week-old spontaneously hypertensive rats (SHR/Izm provided by Disease Model Cooperative Research Association, Kyoto, Japan) bred at Japan SLC, Inc. (Shizuoka, Japan) were kept at the animal laboratory in our institution with a salt load of 1% saltwater and were allowed to eat and drink saltwater ad libitum. Ten-week-old rats were anesthetized with isoflurane followed by intubation using a 16-G indwelling needle with ventilatory respiratory support. We performed LAAE or sham operation using the open intercostal approach in which the pericardium was carefully opened, and the LAA was visualized. The basal side of the LAA was clamped using mosquito tweezers, and the LAA was ligated using 4-0 silk and resected. After resection, the chest was closed. To control perioperative pain, buprenorphine was administered intraperitoneally prior to the procedure, and meloxicam was administered subcutaneously immediately after surgery and on postoperative day (POD) 1. After seven PODs, blood was collected from the abdominal aorta under anesthesia, and rats were sacrificed by injecting a cardioplegic solution (St. Thomas’ No. 2 cardioplegic solution) into the coronary artery through the ascending aorta. Blood pressure was measured at 8, 9, and 10 weeks of age preoperatively and 3 and 7 days postoperatively using the tail cuff method (Softron BP-98A-L, Softron, Tokyo, Japan) with body warming (Softron THC-31, Softron, Tokyo, Japan). Blood was centrifuged for 10 min at 4 °C and at 1,500 × g to obtain the serum, which was separated into cryotubes, frozen in liquid nitrogen, and stored at − 80 °C until further analysis.

### Enzyme-linked immunosorbent assay for ET-1

Using serum samples of rat models, we investigated the concentration of serum ET-1 in each group using the DetectX^®^ Endothelin-1 Enzyme Immunoassay Kit (Arbor Assays, Ann Arbor, MI, USA). Serum samples were handled following the instructions of the manufacturer. Absorbance was measured using the Multiskan SkyHigh microplate reader (Thermo Fischer Scientific, Waltham, MA, USA). The serum concentration of ET-1 in each sample was calculated and compared between the control and the LAAE group.

### Statistical analysis

All statistical analyses were performed using RStudio version 2022.07.2, which was downloaded from Posit website (https://posit.co/products/open-source/rstudio/) based on R 4.2.1 environment, which was downloaded from The Comprehensive R Archive Network (https://cran.r-project.org/). To compare human gene expression between the hypertension and normotension groups, the log_2_ fold change was calculated by subtracting the average of log_2_ (TPM + 1) in the normotension group from that in the hypertension group. In the animal experiment, the blood pressure and heart rate of rat models in the sham and the LAAE groups were compared using exact Wilcoxon rank sum test. The difference of heart rate on POD 0, 3, and 7 were tested using Friedman test. The concentration of serum ET-1 in rat models in the control and the LAAE groups was compared using the Welch’s *t* test. A *p*-value of < 0.05 indicated significance and dismissed the null hypothesis.

## Results

### Patient’s characteristics

The clinical backgrounds of participants are shown in Table 1. AF duration was defined as the period from when AF was first detected to the day of surgery. The LA diameter and ejection fraction were obtained from the preoperative echocardiographic study. There was no significant difference in these parameters between the two groups.

**Table 1:** Patient’s characteristics

	Non-HT	HT	*p* value
*n*	6	6	
Sex M	6	6	
Age (years)	62.0 ± 6.6	68.7 ± 4.4	0.08
BMI (kg/m^2^)	22.8 ± 2.3	24.4 ± 1.7	0.22
AF duration (month)	34.5 ± 64.5	26.0 ± 35.3	0.73
Preop SBP (mmHg)	115.7 ± 6.3	127.3 ± 10.5	0.06
Preop DBP (mmHg)	67.7 ± 4.9	72.3 ± 16.4	0.57
LAD (mm)	49.3 ± 7.0	49.4 ± 4.2	0.99
EF (%)	55.3 ± 5.5	63.0 ± 9.6	0.15
eGFR (mL/min/1.73m^2^)	59.0 ± 13.3	69.4 ± 23.0	0.41
HbA1c (%)	5.8 ± 0.3	5.5 ± 0.3	0.24
BNP (pg/mL)	554.8 (*n* = 3)	227.2 (*n* = 2)	–
NT-proBNP (pg/mL)	610.4 (*n* = 3)	1196 (*n* = 4)	–
Na (mEq/L)	140.6 ± 3.1	139.8 ± 2.8	0.67
K (mEq/L)	4.4 ± 0.4	4.5 ± 0.2	0.81
Cl (mEq/L)	104.4 ± 3.1	105.6 ± 2.7	0.50
FT3 (pg/mL)	3.02 ± 0.36	2.84 ± 1.19	0.41
FT4 (ng/mL)	1.23 ± 0.29	1.26 ± 0.17	0.87
TSH (mIU/mL)	2.78 ± 2.10	2.36 ± 1.19	0.71
ACEi	2	0	0.45
ARB	0	6	< 0.01
Ca antagonist	0	3	0.18
Diuretics	5	2	0.24
Digitalis	1	0	1.00
Beta blocker	4	5	1.00
Statin	2	2	1.00
Antiarrhythmics	0	0	1.00

*HT* hypertension, *BMI* body mass index, *AF* atrial fibrillation, *SBP* systolic blood pressure, *DBP* diastolic blood pressure, *LAD* left atrial diameter, *EF* ejection fraction, *eGFR* estimated glomerular filtration rate, *FT3* free T3, *FT4* free T4, *TSH* thyroid stimulating hormone, *ACEi* angiotensin-converting enzyme inhibitor, *ARB* angiotensin II receptor blocker

### Gene expression profiles

**Fig. 1:**
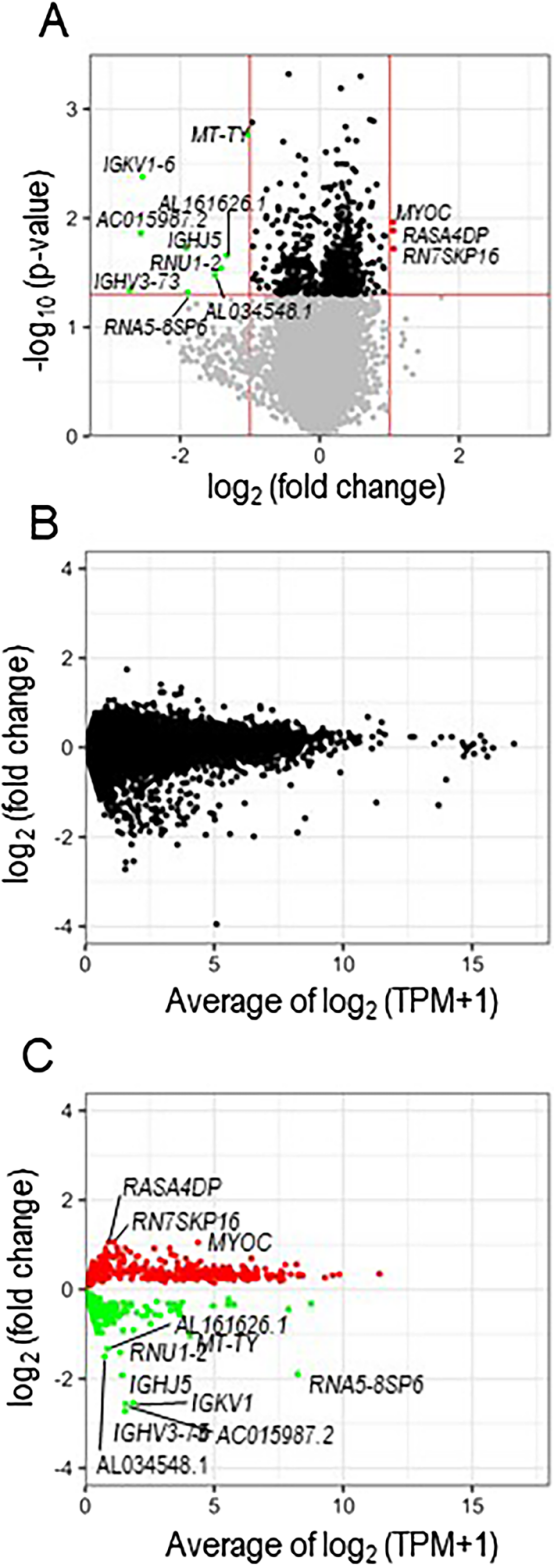
Difference in gene expression profiles in left atrial tissues from the hypertension and normotension groups. (**A**) Volcano plot analysis of global mRNA changes in the hypertension group vs. normotension group. Each circle represents one mRNA; red circles are upregulated genes, and green circles are downregulated genes. The Y-axis shows the − log_10_ values of the *p*-value. *p* values (0.05) and fold changes (2, 1/2) are indicated by red lines. (**B**) MA plot analysis of global mRNA changes in the hypertension group vs. normotension group. The x-axis denotes the average of log_2_ expression value across samples normalized by transcripts per million, and the y-axis denotes the log_2_ fold change in the given contrast. (**C**) MA plot analysis of the selected mRNAs that were significantly upregulated (red circles) and downregulated (green circles) in the hypertension group (*p* < 0.05)

We applied RNA-Seq technology to profile the transcriptome of the LAA of hypertensive and normotensive patients. Overall, 57,773 raw reads were produced from the platform, and 36,002 (62.3%) clean reads were generated after trimming the adaptor sequences. A modified volcano plot analysis was applied to identify the RNA differentially expressed in the LAA from hypertensive patients (Fig. 1A). When the threshold of log_2_ fold change was set to > 1 with a *p*-value of < 0.05, there were three and nine up- and downregulated genes, respectively. Figure 1B shows these data transformed into M (log ratio) and A (average) scales or “minus over average” (MA) plot. The MA plot is an application of the Bland–Altman plot that is a visual representation of genomic data as well as visualizes differences between measurements from two samples. Although MA plots are originally applied for two-channel DNA microarray gene expression data, they may also be used to visualize high-throughput sequencing analysis [7, 8]. Figure 1C exhibits a replotting of data using a *p*-value of < 0.05. Although RNA5-8SP6 was highly expressed and significantly downregulated, it may not have an impact on hypertension as it is a pseudogene. Additionally, other genes may not have important roles in hypertension as their expression levels were low.

### Multivariate analysis

**Fig. 2:**
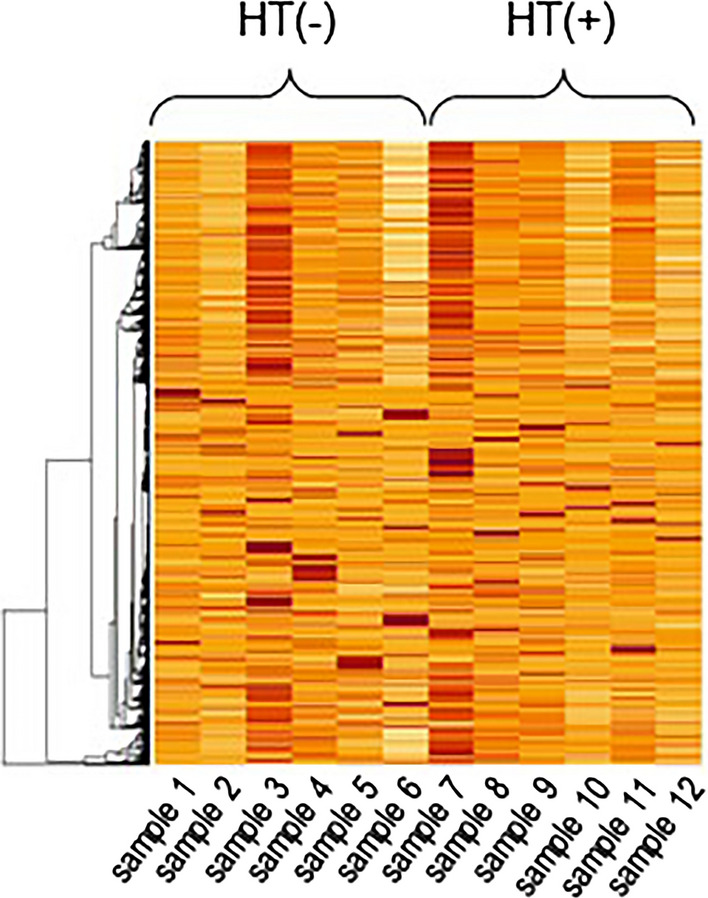
Heatmap of all expressed genes in each sample (samples 1–6 and 7–12 were from patients without and with hypertension, respectively). Rows, mRNAs; columns, patient number. Color ranges: higher, median, and lower expression is represented by red, orange, and yellow, respectively

**Fig. 3:**
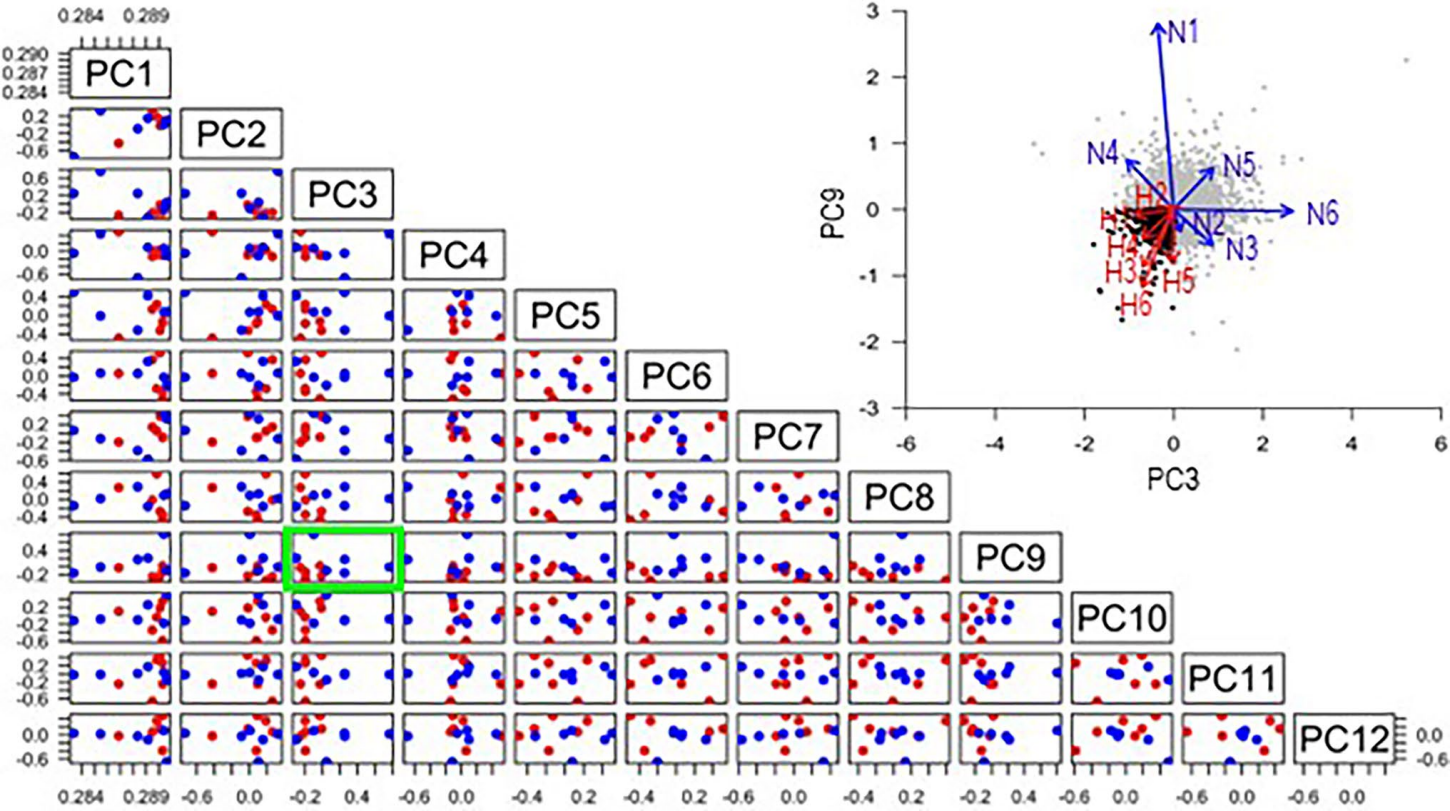
Principal component (PC) analysis to detect differences in segmental coordination between the hypertension and normotension groups. Red and blue circles represent PC vectors of the hypertension and normotension groups, respectively. In the combination of PC3 and PC9 (green square), vectors of the hypertension group gather in PC3 < 0 and PC9 < 0. In inset, PC scores of expressed genes in PC3 and PC9 are shown with PC vectors overlapping by arrows, where genes with scores of PC3 < 0 and PC9 < 0 (shown as black circles) were extracted for pathway analysis

As gene identification results for hypertension based on the volcano and MA plots were less informative, we then applied hierarchical clustering using LAA samples of the 12 patients. Hierarchical clustering is a method of cluster analysis that creates a hierarchical representation of clusters in a dataset. Unexpectedly, hierarchical clustering failed to identify groups of hypertension-specific genes (Fig. 2). Hence, the need for more reliable analytical tools motivated us to apply PCA. PCA is one of the most used dimensional reduction techniques in biological investigations. Gene expression in individual human is usually affected by a lot of factors such as familial background, basic diseases, medication, lifestyle, and so on. PCA can make it possible to reduce the dimension of expression data, to visualize the similarities between the biological samples, and to filter noise, which leads us to discovering the characteristics of the gene expression pattern among the group of our interest in the RNA-Seq result affected by these multiple factors. Our approach was to perform PCA on RNA-Seq data (Fig. 3). Each sample had a vector based on the tendency of gene expression levels in the first to the twelfth principal component. In the combination of the third (PC3) and ninth principal components (PC9), the hypertension group gathered in PC3 < 0 and PC9 < 0. Genes that had a principal component score in this area were extracted for pathway analysis.

### Pathway analysis

**Fig. 4:**
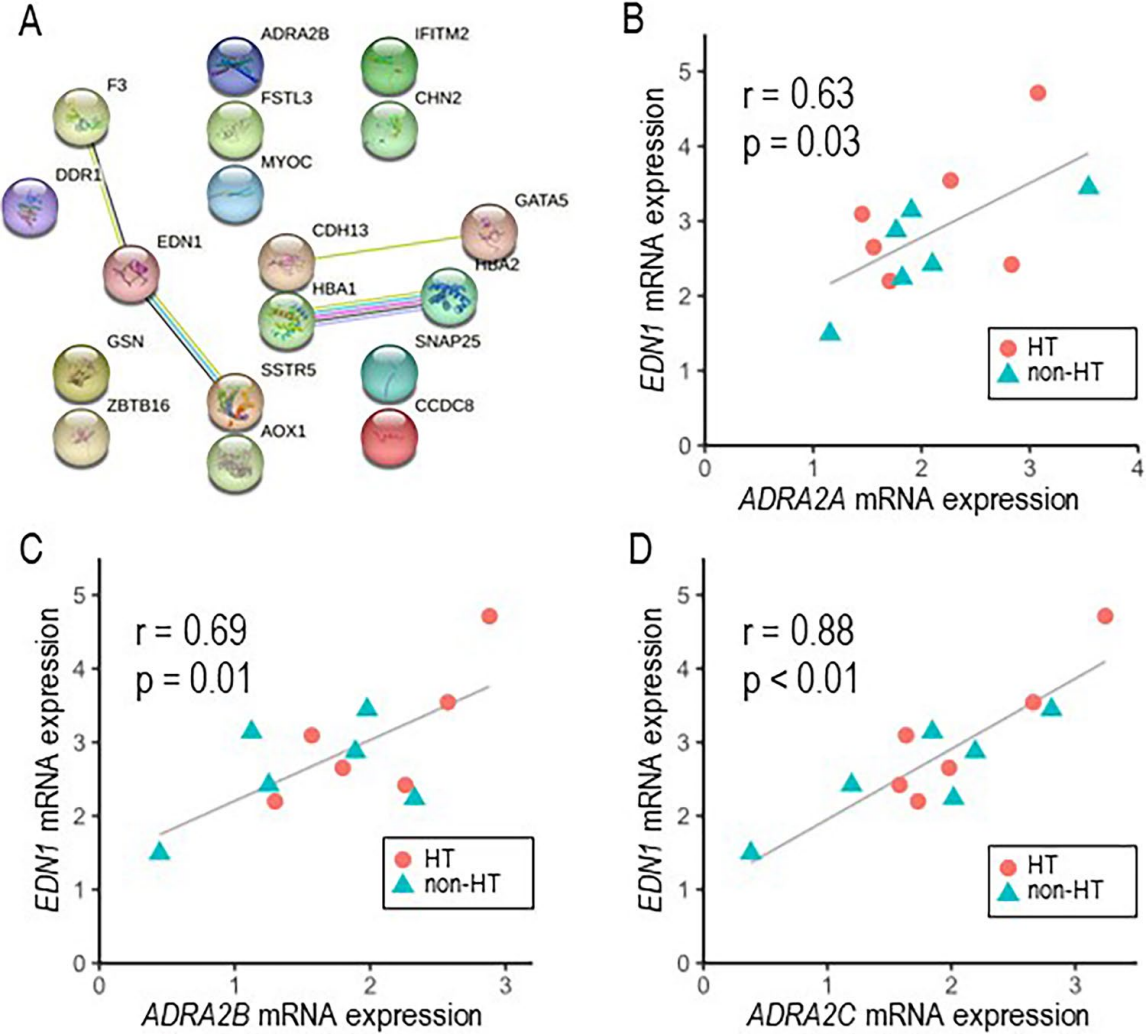
STRING analysis of predicted function partners for genes in the left atrial appendage of patients with hypertension. (**A**) Known interactions are shown in cyan lines (database) and in magenta lines (experimentally determined). Predicted interactions are shown in blue lines (gene co-occurrence). Others are shown in yellow lines (text-mining), in black lines (co-expression), and in purple lines (protein homology). (**B**, **C**, **D**) Association of the expression levels of *EDN1* with *ADRA2A* (B), *ADRA2B* (**C**), and *ADRA2C* (**D**) with linear-regression analysis. Red circles for hypertension, and blue triangles for normotension patients

The main goal of pathway analysis is to identify a statistically significant association between biologically related genes and a target phenotype. Therefore, the way genes are grouped will determine the conclusions drawn from the analysis. To upload data analysis results from this experiment, IPA was applied accordingly. IPA is a web-based bioinformatics application that identifies data sets for functional analysis, integration, and further understanding [9]. Eighteen genes associated with hypertension were identified by the downstream analysis “diseases and functions” in IPA. These genes include *ADRA2B*, *AOX1*, *CCDC8*, *CDH13*, *CHN2*, *DDR1*, *EDN1*, *F3*, *FSTL3*, *GATA5*, *GSN*, *HBA1*, *HBA2*, *IFITM2*, *MYOC*, *SNAP25*, *SSTR5*, and *ZBTB16* (Table 2). Among them, *ADRA2B* and *EDN1* encode the α2B adrenergic receptor (AR) and ET-1, respectively, both of which produce vasoconstriction. A system-wide understanding of how these genes function requires knowledge of all functional interactions between the expressed proteins. The STRING database aims to collect and integrate this information by consolidating known and predicted protein–protein association data for many organisms. By using STRING analysis on these 18 hypertension-associated genes, we successfully demonstrated a possible functional association between them (Fig. 4A). The STRING database contains information from numerous sources shown by different color lines; cyan lines were drawn according to the results by (database), magenta lines were by (experimentally determined), blue lines were by (gene co–occurrence), yellow lines were by (text–mining), black lines were by (co–expression), and purple lines were by (protein homology). α2 AR (α2-AR) has 3 subtypes including, α2a-AR (*ADRA2A*), α2b-AR (*ADRA2B*) and α2c-AR (*ADRA2C*). Interestingly, expression levels of *ADRA2A* (*r* = 0.63, *p* =   0.03), *ADRA2B* (*r* = 0.69, *p* = 0.01) and *ADRA2C* (*r* = 0.88, *p* < 0.01) significantly and positively correlated with that of *EDN1* (Fig. 4B–D), although STRING analysis failed to specify the possible genetic interaction between α2 adrenergic receptors (ADRA2) and endothelin-1.

**Table 2:** Hypertension-associated genes expressed in the LAA

Gene symbol	Gene full name	Fold change	*p* value
*ADRA2B*	adrenoreceptor alpha 2B	1.47	0.01
*AOX1*	aldehyde oxidase 1	1.63	0.15
*CCDC8*	Coiled-coil domain containing 8	1.42	0.03
*CDH13*	cadherin 13	1.48	0.05
*CHN2*	chimerin 2	1.68	0.05
*DDR1*	discoidin domain receptor tyrosine kinase 1	1.45	0.05
*EDN1*	Endothelin-1	1.42	0.32
*F3*	coagulation factor III, tissue factor	1.51	0.13
*FSTL3*	Follistatin-like 3	1.46	0.28
*GATA5*	GATA-binding protein 5	1.53	0.04
*GSN*	gelsolin	1.49	0.08
*HBA1*	hemoglobin subunit alpha 1	2.65	0.27
*HBA2*	hemoglobin subunit alpha 2	2.52	0.17
*IFITM2*	Interferon-induced transmembrane protein 2	1.43	0.28
*MYOC*	myocilin	2.07	0.01
*SNAP25*	Synaptosome-associated protein 25	1.44	0.23
*SSTR5*	somatostatin receptor 5	1.44	0.37
*ZBTB16*	zinc finger and BTB domain containing 16	1.42	0.12

### Effect of LAAE on blood pressure and serum ET-1 concentration in rats

**Fig. 5:**
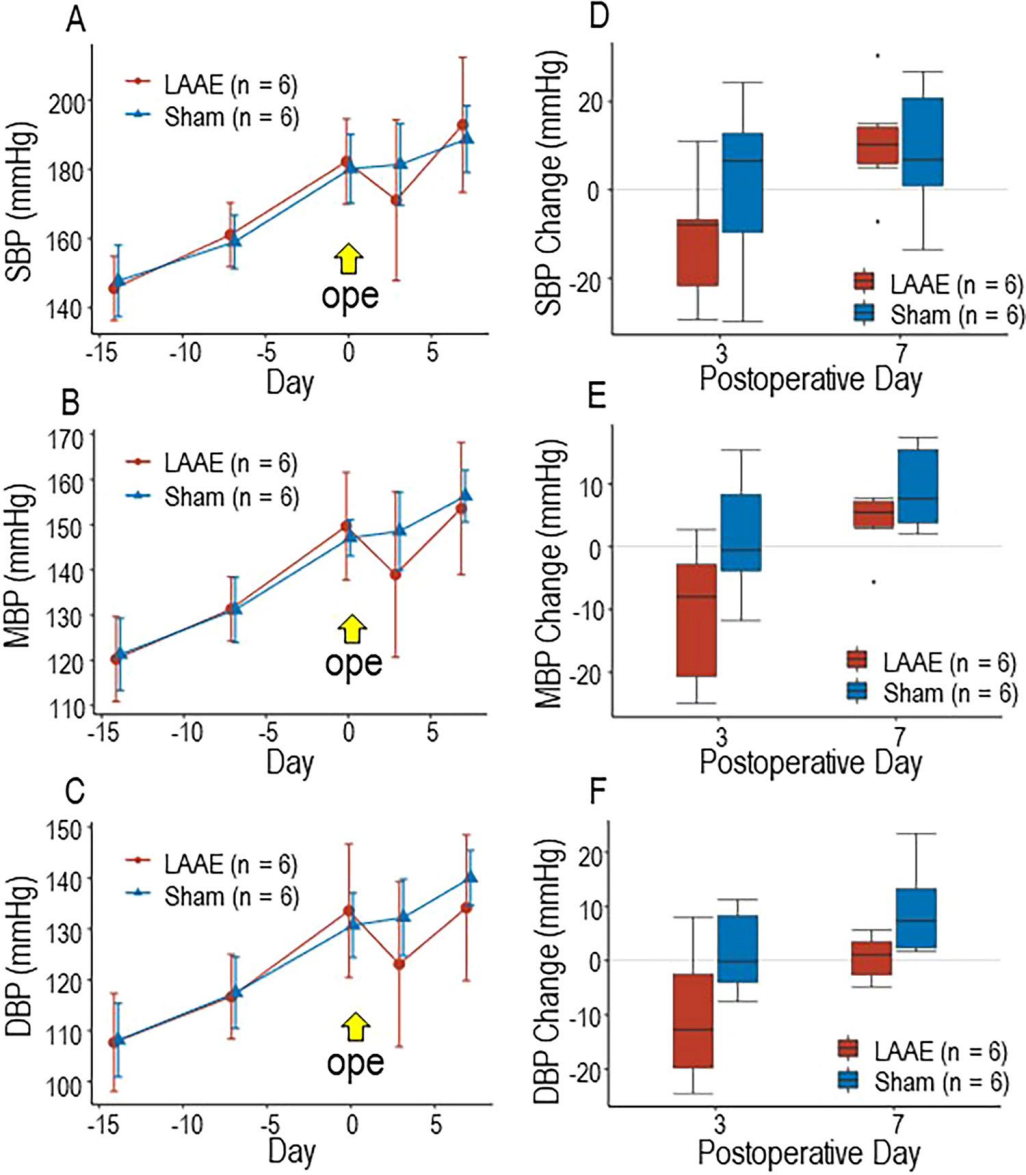
Blood pressure changes after LAAE or sham operation in SHR. (**A**, **B**, **C**) Daily changes in systolic blood pressure (SBP) in (**A**), mean blood pressure (MBP) in (**B**), and diastolic blood pressure (DBP) in (**C**) before and after LAAE. Error bar of each plot indicates the range of standard deviation. (**D**, **E**, **F**) Postoperative change in SBP (**D**), MBP (**E**), and DBP (**F**) at day 3 and day 7 from baseline in each group. The horizontal line of the box indicates the median. The upper and lower edge of the box indicates the upper and lower quartiles, respectively. The upper and lower whisker indicates maximum and minimum, respectively

**Fig. 6:**
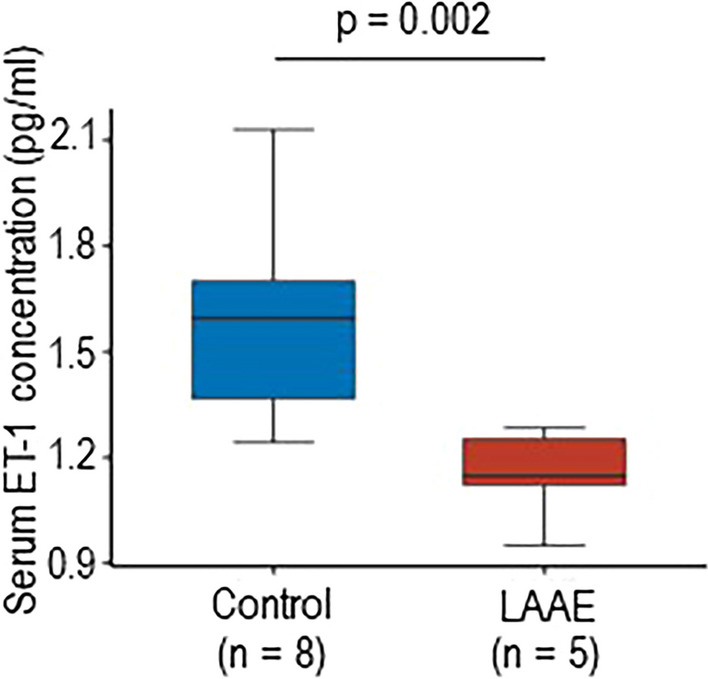
Changes in serum ET–1 concentration after LAAE. Serum ET–1 concentrations were measured in SHR rats in the control (*n* = 8) and after LAAE on 7 POD (*n* = 5). The horizontal line of the box indicates the median. The upper and lower edge of the box indicates the upper and lower quartiles, respectively. The upper and lower whisker indicates maximum and minimum, respectively

To further confirm the role of atrial ET-1 in the regulation of blood pressure or systemic arterial pressure, an animal model was developed to test the hypothesis that LAAE improves blood pressure due to a reduction in serum ET-1 concentration. Figure 5 demonstrates changes in the systolic, mean, and diastolic blood pressures of rats in the LAAE and sham groups. Blood pressure in the LAAE group was appreciably reduced on POD 3 compared to that of the sham group, and the reduction in diastolic blood pressure was maintained in the LAAE group on POD 7. Diastolic blood pressure in the LAAE group was lower by 9.2 mmHg assessed by the average blood pressure or by 12.6 mmHg assessed by the median blood pressure changes than those in Sham group on POD 3. Although the difference of blood pressure between the groups became narrower, diastolic blood pressure in the LAAE group was still lower by 5.9 mmHg assessed by the average blood pressure or by 6.2 mmHg assessed by the median blood pressure change than those in Sham group on POD 7 (Fig. 5C and F). The heart rate (HR) of both groups did not significantly change postoperatively (*p* = 0.11 in Sham group, *p* = 0.61 in LAAE group, Suppl. Figure). There is no significant difference between LAAE and Sham groups in the preoperative HR (*p* = 0.24) and in the HR on POD 3 (*p* = 0.82) and POD7 (*p* = 0.31). More importantly, serum ET-1 concentrations in LAAE rats were significantly lower than those in the control group (without LAAE operation), suggesting the importance of LAA-derived ET in blood pressure regulation (Fig. 6).

## Discussion

**Fig. 7:**
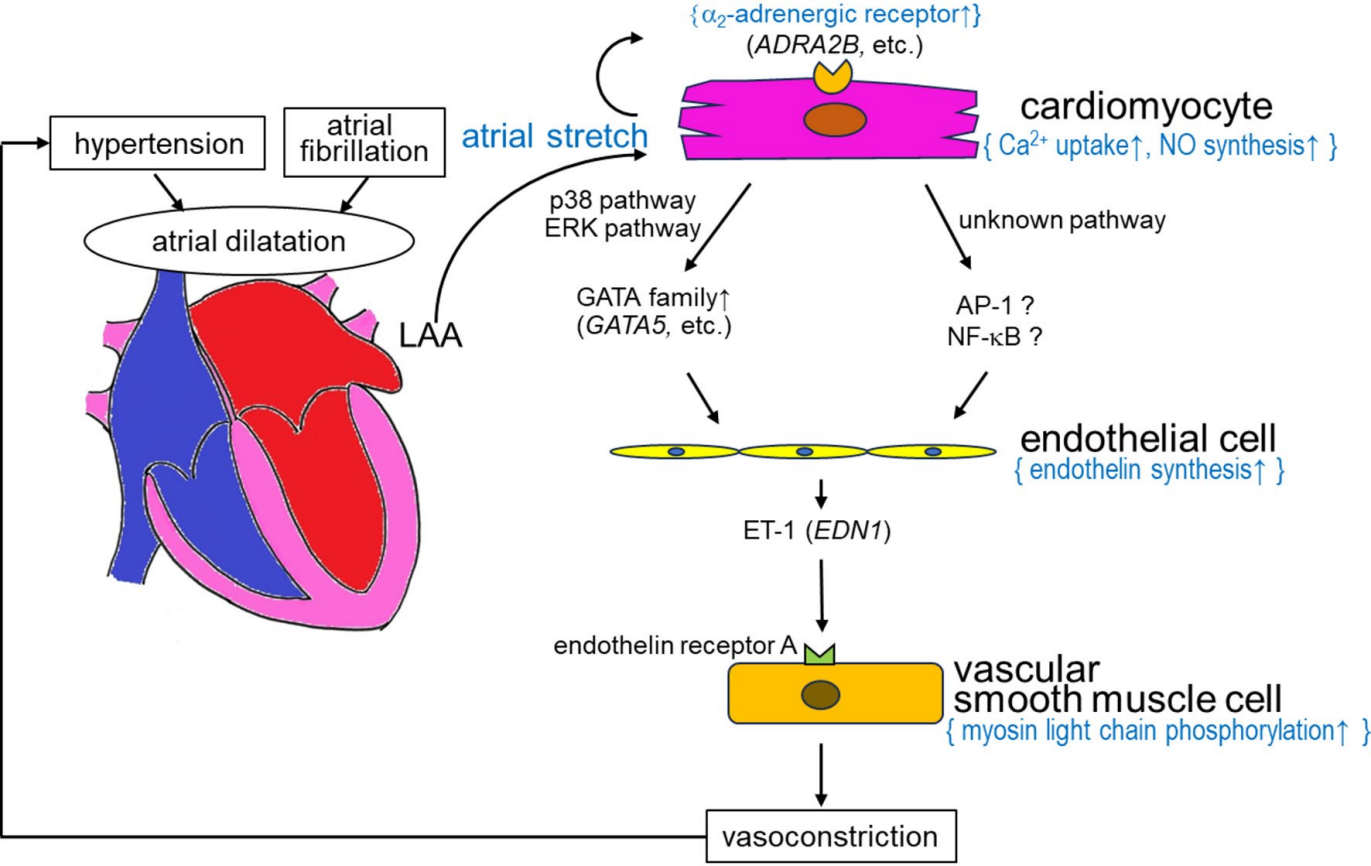
Visualized summary of the result demonstrating a role of atrial endothelin-1 as a potential factor responsible for blood pressure regulation. Resection of the atrial appendage may improve high blood pressure accompanied by a decrease in serum endothelin-1 concentration possibly upon relaxation of vasoconstriction

The main finding of this study is that atrial ET-1 may be associated with blood pressure improvement after LAA resection in patients with hypertension. Additionally, aside from the *EDN1* or ET-1 gene, several genes in the atrium, including *ADRA2B*, *AOX1*, *CCDC8*, and *CDH13,* may be involved in blood pressure regulation. Figure 7 presents a graphical conclusion for the role of atrial endothelin-1 as a potential factor responsible for blood pressure regulation.

Little is known about the regulatory function of the atrium on systemic blood pressure. Recent studies have demonstrated that LAAE using the Lariat device in patients with AF resulted in a sustained decrease in blood pressure [5, 6, 10]. A decrease in serum concentrations of aldosterone and catecholamines after the Lariat procedure may also account for this phenomenon [10]. These clinical reports motivated us to investigate the mRNA expression profiles in the LAA of patients with hypertension and AF. Combined with PCA and pathway analysis, we demonstrated that *EDN1* expression, which codes for ET-1, was highly expressed in the atrium of patients with hypertension than that of patients without hypertension (fold change = 1.42, *p* = 0.32), suggesting the involvement of atrial ET-1 in blood pressure regulation. Meanwhile, the expressions of angiotensinogen and angiotensin-converting enzyme were not significantly increased (fold change = 1.11, *p* = 0.61; fold change = 1.23, *p *= 0.23, respectively). Combined with the understanding that ET-1 is expressed in the atria of SHR and DOCA-salt-induced hypertension rats more than in those of WKY [11, 12], and that hypertension is one of the significant factors for the square root of ET-1 expression in the human LAA [13], atrial expression of the ET-1 gene could play a role in blood pressure regulation in humans and rats.

ET-1 is a vasoconstrictive factor that is mainly produced in the vascular endothelium, and its receptors are ETA and ETB [14]. The ETA receptor is abundantly expressed in vascular smooth muscles, whereas the ETB receptor is expressed in both vascular smooth muscles and vascular endothelium [14]. The ETA receptor exhibits vasoconstrictor activity through ET-1 stimulation, whereas the ETB receptor also exhibits vasoconstrictor activity on vascular smooth muscles. In contrast, the ETB receptor on the endothelium exhibits vasodilatory activity by releasing NO and prostacyclin [15–17]. ET-1 is also abundant in pulmonary vessels and plasma in patients with pulmonary hypertension [18]. Hence, ET-1 receptor antagonists are often used to treat pulmonary hypertension [18]. Meanwhile, the functional significance of atrial ET-1 in patients with essential hypertension remains unclear, although some studies revealed elevated plasma ET-1 concentrations in patients with essential hypertension [19, 20]. Interestingly, normotensive subjects with high plasma ET-1 concentrations have an increased risk of developing hypertension [21]. Taken together, surgery-mediated reduction in plasma ET-1 concentrations may account for the regulation of blood vessel tone and blood pressure in patients with or without AF.

Atrial dilatation or atrial muscle stretching is one of the important causes of AF [22, 23]. Atrial stretching increases mRNA expression of atrial ET-1 (*EDN1*) [24] and ET-3 (*EDN3*) [23]. The GATA family is a well-known transcription factor of *EDN1*. Through mechanical stretching, DNA binding of *GATA4* was upregulated in isolated atria of rats via p38 and extracellular signal-regulated protein kinase pathways [25]. Among the GATA family, *GATA4*, *GATA5*, and *GATA6* are mainly expressed in the heart [26]. In this study, *GATA5* was extracted as one of the hypertension-associated genes in the atrium (fold change = 1.53, *p* = 0.04). *GATA4* expression also tended to be higher in the hypertension group, although its fold change was lower than that of *GATA5* (fold change = 1.33, *p* = 0.06). This result indicates that atrial stretching caused by hypertension may increase the expression of not only ET-1 but also of the GATA family of genes, further increasing ET-1expression. Except for the GATA family, several transcription factors may account for ET-1 expression, including vascular endovascular zinc finger 1 (*VEZF1*), forkhead box O (*FOXO*), AP-1 (*JUN* and *FOS*), hypoxia inducible factor 1 (*HIF1A*), Smad2, Smad3, Smad4, and NF-kB [27]. However, expression of these genes did not significantly change in the atrium of patients with hypertension, although their upstream signal molecules were not studied.

While the current experiment revealed that ET-1 is likely to be responsible for blood pressure regulation, pathway analysis also indicated a possible involvement of the α2-AR. The α2-AR regulates cardiac left ventricular peak systolic and diastolic pressure as well as right atrial pressure [28]. α2B-AR as well as α2A-AR are highly expressed in cardiomyocytes of SHR [29]. Activation of the α2-AR signaling pathway can promote NO synthesis and reuptake of intracellular Ca^2+^ into the sarcoplasmic reticulum, thus suppressing the increase in intracellular Ca concentration [30]. As NO-mediated Ca reuptake is impaired in SHR from a young age, it is likely that upregulated α2-AR in the myocardium promotes Ca reuptake and exerts a protective effect on the myocardium [30]. Transcriptional activity of transcription factors AP-1 and NF-kB was upregulated by adrenergic stimulation in a rat pheochromocytoma cell line expressing a2A/B/C-AR [31]. As AP-1 and NF-kB are dominant transcription factors of ET-1, increased α2-AR expression could be involved in increased ET-1 expression, although the expression of AP-1 and NF-kB mRNAs did not significantly change in the LAA of patients with hypertension in this study. Alternatively, a significant correlation between α2-AR (*ADRA2A*, *ADRA2B*, and *ADRA2C*) and ET-1 (*EDN1*) expression levels (*ADRA2A*: *r* = 0.63, *p* = 0.03, *ADRA2B*: *r* = 0.69, *p* = 0.01, *ADRA2C*: *r* = 0.88, *p* < 0.01) (Fig. 4B–D) was identified. These observations may indirectly support the possible relationship between α2-AR and ET-1 expression.

The limitations of this study are that all human LAA samples were obtained from patients with AF because of ethical obligations. AF leads to atrial myocardium thickness, fibrosis, and remodeling, which can affect gene expression [32–34]. Structural heart diseases, including valvular diseases, are also responsible for cardiac ventricular and atrial remodeling. As this investigation enrolled multiple patients suffering from these diseases, unspecified factors may account for the results. Additionally, all patients were treated with multiple medications, including angiotensin II receptor blockers; the impact of these medications on the results should also be accounted for. As it was not ethically acceptable to measure plasma ET-1 levels in these patients, the direct evidence of the correlation between left atrial appendectomy and changes in plasma ET-1 concentrations was not precisely elucidated. Furthermore, results examined by comprehensive RNA-seq methods for transcriptome analysis were not reconfirmed by RT-PCR analysis. Further investigations are needed to clarify the mechanisms responsible for blood pressure regulation in humans following LAAE.

## Declarations

### Data availability

Data in this study are available from the corresponding author on reasonable request.
